# DNA and RNA oxidative damage in the retina is associated with ganglion cell mitochondria

**DOI:** 10.1038/s41598-022-12770-9

**Published:** 2022-05-24

**Authors:** Lei Gu, Jacky M. Kwong, Joseph Caprioli, Natik Piri

**Affiliations:** 1 Stein Eye Institute, University of California, Los Angeles, 100 Stein Plaza, Los Angeles, CA 90095 USA; 2 Brain Research Institute, University of California, Los Angeles, Los Angeles, CA 90095 USA

**Keywords:** Cell biology, Molecular biology, Neuroscience

## Abstract

This study examines retinas from a rat glaucoma model for oxidized nucleosides 8OHdG and 8OHG, biomarkers for oxidative damage of DNA and RNA, respectively. Immunohistochemical data indicate a predominant localization of 8OHdG/8OHG in retinal ganglion cells (RGCs). The levels for these oxidized DNA/RNA products were 3.2 and 2.8 fold higher at 1 and 2 weeks after intraocular pressure elevation compared to control retinas, respectively. 8OHdG/8OHG were almost exclusively associated with mitochondrial DNA/RNA: ~ 65% of 8OHdG/8OHG were associated with RNA isolated from mitochondrial fraction and ~ 35% with DNA. Furthermore, we analyzed retinas of the rd10 mouse, a model for retinitis pigmentosa, with severe degeneration of photoreceptors to determine whether high levels of 8OHdG/8OHG staining intensity in RGCs of control animals is related to the high level of mitochondrial oxidative phosphorylation necessary to support light-evoked RGC activity. No significant difference in 8OHdG/8OHG staining intensity between control and rd10 mouse retinas was observed. The results of this study suggest that high levels of 8OHdG/8OHG in RGCs of wild-type animals may lead to cell damage and progressive loss of RGCs observed during normal aging, whereas ocular hypertension-induced increase in the level of oxidatively damaged mitochondrial DNA/RNA could contribute to glaucomatous neurodegeneration.

## Introduction

Oxidative stress occurs when the generation of reactive oxygen species (ROS) exceeds the ability of cells to neutralize and eliminate them. The excess of free radicals, and ROS in particular, can damage cellular constituents and initiate the apoptotic signaling pathway^1,2^. Oxidative stress has been implicated in many age-related neurodegenerative diseases, including glaucomatous optic neuropathy^3–6^, which affects millions of people worldwide and is often diagnosed after patients have suffered irreversible damage to their vision. If left untreated, the disease can lead to debilitating visual impairment^7,8^. Vision loss in glaucoma is due to damage to retinal ganglion cells (RGCs) and their axons in the optic nerve^9–11^. RGCs’ susceptibility to damage in glaucoma is believed to be associated with multiple risk factors. These factors determine the likelihood of developing the disease, as well as its severity and rate of progression. Some of the cellular factors that are commonly associated with the pathogenesis of glaucoma include biomechanical stress associated with intraocular pressure (IOP), axonal transport failure, insufficient nutrient supply, autoimmunity, glial cell dysfunction and oxidative stress, which has been associated with mitochondrial dysfunction^3–5^. However, the precise molecular mechanisms are not well understood and may be different in different forms of the disease. Our analysis of retinal gene expression profiles of animal glaucoma models implicated the proteins of the thioredoxin (Trx) system in the elevated IOP-induced oxidative process. The Trx system is a ubiquitous thiol-reducing antioxidant system that includes Trx, Txnip, Trxr (Trx reductase) and NADPH^12,13^. Trx proteins—cytoplasmic Trx1 and mitochondrial Trx2—protect cells from oxidative stress by scavenging for intracellular ROS^14^. The oxidized Trx can be converted back to its reduced form by Trxr in the presence of NADPH. Txnip, on the other hand, can bind to the Trx active site and negatively regulate its reducing activity^15^. In glaucomatous retinas, we observed a significant upregulation of Txnip at both mRNA and protein levels, which in turn can diminish Trx ROS-neutralizing activity and lead to oxidative damage. To test this hypothesis, we overexpressed Trx1 and Trx2 in glaucomatous retinas and, as expected, this resulted in significant increase in survival of RGCs injured by elevated IOP^16^. These data suggest that dysregulation of the Trx system can compromise RGC redox state and consequently, their function and integrity. The Trx system was also implicated in human glaucoma. A meta-analysis of eight independent genome-wide association studies identified Trx reductase 2 (TXNRD2) as a susceptibility locus for primary open-angle glaucoma^17^. Expression of this gene is enriched in RGCs and astrocytes of the optic nerve head, suggesting that mutations in TXNRD2 could lead to oxidative stress and RGC apoptosis in glaucoma^17^.

The involvement of oxidative stress in the pathogenesis of neurodegenerative diseases is generally associated with oxidized cellular macromolecules, including proteins, lipids, DNA and RNA. In this current study, we examined retinal tissues from a rat experimental model of glaucoma for 8-hydroxydeoxyguanosine (8OHdG) and 8-hydroxyguanosine (8OHG), which are the biomarkers for oxidative damage of DNA and RNA, respectively. Immunohistochemical analysis of mouse and rat retinas indicate predominant localization of 8OHdG/8OHG in RGCs. These oxidized products were associated with mitochondrial DNA/RNA. The intensity of 8OHdG/8OHG staining was significantly higher in glaucomatous retinas both at 1 and 2 weeks after IOP elevation than that of control tissues. This observation was corroborated by quantitative analysis of 8OHdG/8OHG level in retinas of experimental animals. We also speculated that the observed high level of 8OHdG/8OHG immunostaining intensity in RGCs compared to other retinal cells in wild-type animals may be associated with the functions of these cells, particularly light-evoked activity, that demands high levels of mitochondrial activity. To address this possibility, we evaluated retinas of retina degeneration 10 (rd10) mice^18^ that sustained near complete photoreceptor loss. The rationale for using rd10 animals was based on the assumption that in retinas with severe degeneration of photoreceptors, the high energy-demanding function of RGCs associated with generation and propagation of action potentials along the axons to the targets in the brain, with the exception of a very small population of intrinsically photosensitive RGCs, will be dramatically diminished. This will decrease the need for ATP/oxidative phosphorylation (OXPHOS) and accompanying this process, production of free radicals that are responsible for oxidative modification of DNA/RNA. However, no significant change in 8OHdG/8OHG immunostaining intensity in RGCs of these rd10 animals compared control mice was found.

## Results

### Oxidative modification of DNA/RNA in the retinas of the rat glaucoma model

**Figure 1:**
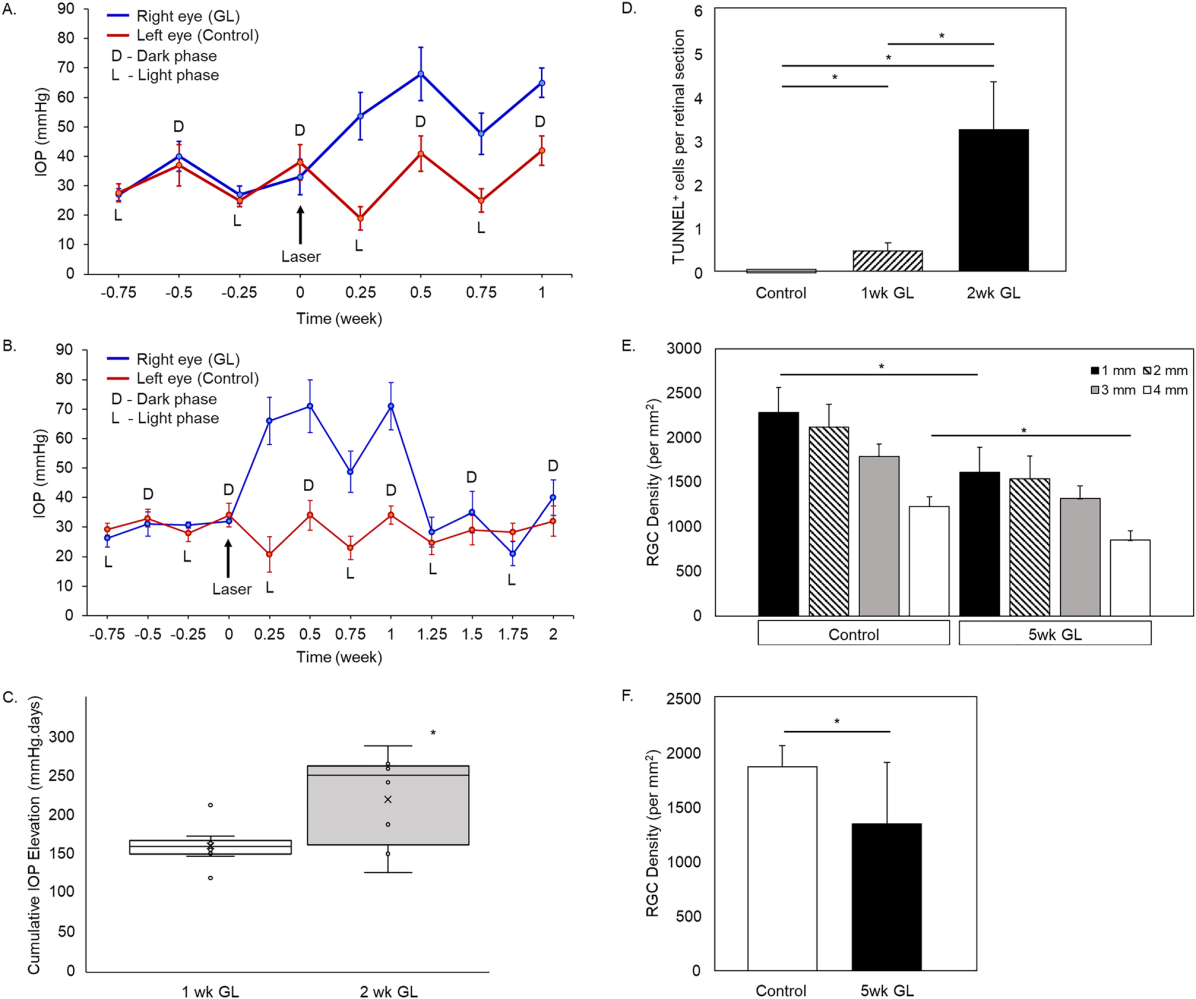
The light and dark phase IOP profiles for laser-treated and contralateral control eyes. Trabecular laser photocoagulation resulted in a significant increase of IOP during 1 (**A**; n = 10) and 2 (**B**; n = 10) weeks after the procedure compared to untreated contralateral eyes. IOP profiles of representative animals of 1 and 2 weeks after laser photocoagulation were shown. RE, right eye; LE, left eye. (**C**) Comparison of cumulative IOP elevation at 1 week and 2 weeks after trabecular laser photocoagulation. There is a significant increase in cumulative IOP elevation at 2 weeks after photocoagulation compared to 1 week group (n = 10; *P* = 0.012). Each circle in the bar chart represents individual data points. (**D**) Quantification of TUNEL-positive RGCs in control and glaucomatous retinas 1 and 2 weeks after IOP elevation. TUNEL staining was performed on retinal sections from control (n = 6) and experimental eyes 1 (n = 3) and 2 (n = 3) weeks of IOP elevation. The number of TUNEL-stained ganglion cells per eye was determined from at least 6 representative sections. TUNEL-positive cells were detected in glaucomatous retinas 1 and 2 weeks after IOP elevation, but not in control retinas. The difference in the number of TUNEL-positive cells between eyes with elevated IOP and controls eyes was statistically significant (*P < 0.05). E. RGC loss 5 weeks after IOP elevation. Approximately 30% of cell loss in experimental retinas (n = 8) compared to controls (n = 8) was observed: 29.4% (*P* = 0.023), 27.3% (*P* = 0.053), 26.5% (*P* = 0.061) and 30.8% (*P* = 0.022) RGC loss at 1, 2, 3 and 4 mm from the center of the optic nerve, respectively (**E**). (**F**) Average RGC density 5 weeks after trabecular laser photocoagulation. There was a significant reduction in the average RGC density for the entire retinas after 5 weeks of IOP elevation compared to controls (*P* = 0.035). Data are presented as mean $\pm$ SD. 1 wk GL, 1 week glaucoma; 2 wk GL, 2 weeks glaucoma; 5 wk GL, 5 week glaucoma.

**Figure 2:**
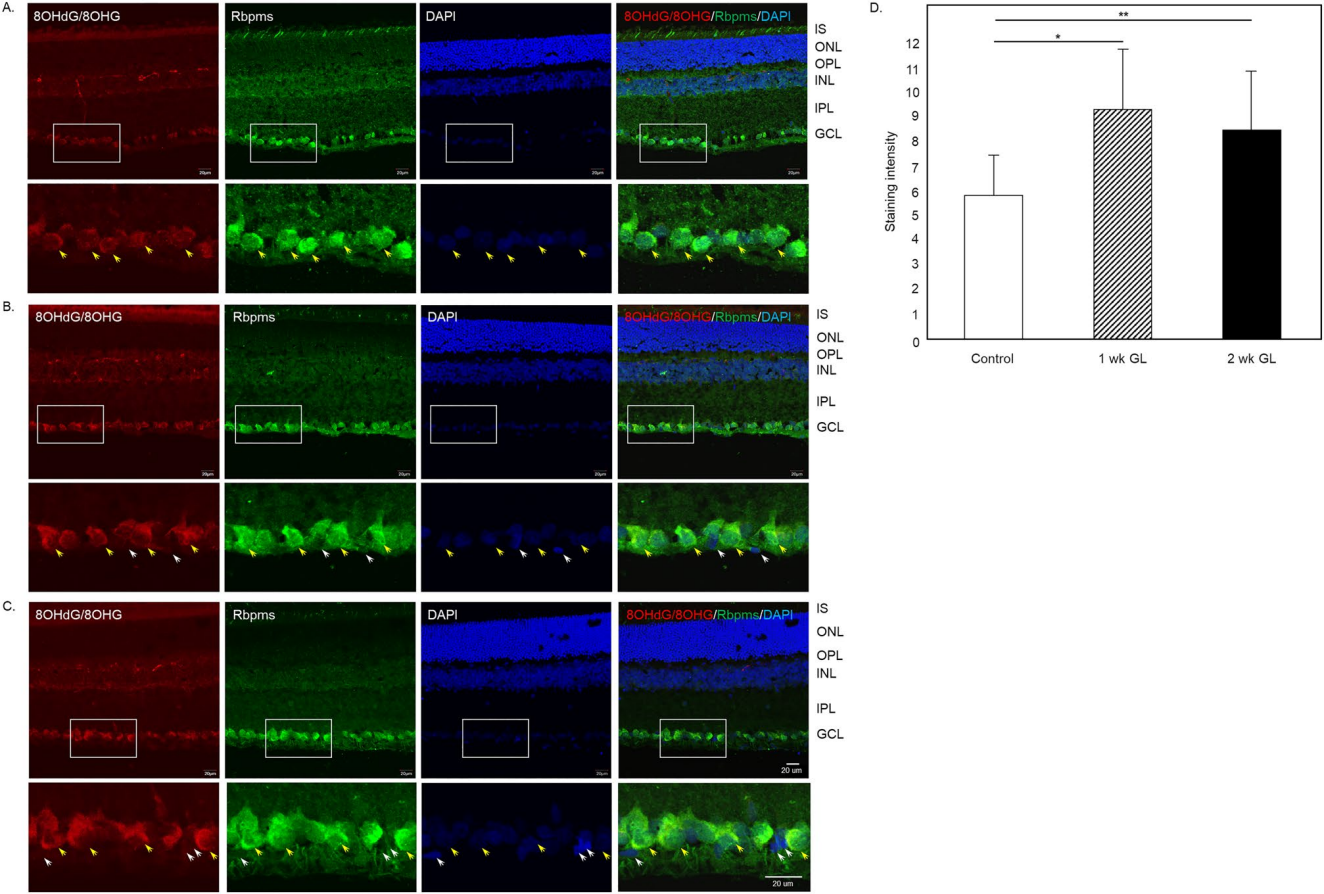
DNA/RNA oxidative damage in retinas of experimental glaucoma model. Immunostaining of retinal sections with monoclonal antibodies against for 8OHdG/8OHG, markers of oxidative damage to DNA and RNA, showed a strong reaction with cells within GCL of control (**A**) and glaucomatous retinas 1 (**B**) and 2 (**C**) weeks after IOP elevation. These 8OHdG/8OHG-positive cells were RGCs as they were stained with an RGC marker Rbpms (yellow arrows). Rbpms-negative cells in the GCL were also negative or poorly stained for 8OHdG/8OHG (white arrows). Although RGCs in both control (**A**) and hypertensive retinas (**B** and **C**) were 8OHdG/8OHG-positive, it appears that the intensity of staining in glaucomatous retinas is higher than in the controls. DAPI was used as a nuclear counterstain. (**D**) Quantitative analysis of RGC immunoflorescence in control and experimental animals 1 week and 2 weeks after IOP elevation. Ninety six RGCs from each group (n = 4 per group) were analyzed. Staining intensities in control, 1 and 2 weeks glaucomatous retinas were 5.56 ± 1.74, 9.24 ± 2.60 and 8.22 ± 2.69, respectively. The difference in RGC staining intensity between experimental and control retinas was statistically significant: *P = 4.68252E-20 and **P = 2.50218E-12. Data are presented as the mean ± SD. IS, photoreceptor inner segments; ONL, outer nuclear layer; OPL, outer plexiform layer; INL, inner nuclear layer; IPL, inner plexiform layer; GCL, ganglion cell layer; 1 wk GL, 1 week glaucoma; 2 wk GL, 2 weeks glaucoma.

A rat glaucoma model was generated by moderate, chronic IOP elevation. The model has been well characterized and extensively used by us and others^19–26^. Since oxidative stress is known to be a relatively early event that takes place during elevated IOP-induced glaucomatous RGC degeneration, we investigated changes induced by IOP elevation at 1 and 2 weeks post-laser treatment. The average light phase and dark phase IOP readings before and after trabecular laser photocoagulation are presented in Table 1. Significant differences of both light and dark phases IOP in the laser treatment group compared to the corresponding control were observed at 1 week and 2 weeks after laser treatment (Table 1; *P* < 0.05). The changes of IOP profile in experimental eyes at 1 week (Fig. 1A) and 2 weeks (Fig. 1B) after laser treatment were remarkable when compared to contralateral control eyes. The average cumulative IOP elevation in experimental eyes was 158.6 ± 23.4 mm Hg.days and 217.6 ± 68.8 mm Hg.days for 1 week (n = 10) and 2 weeks (n = 10) after laser-treatment respectively when compared to contralateral control eyes (Fig. 1C). There was a significant increase in cumulative IOP elevation from 1 to 2 weeks after laser-treatment (*P* = 0.012). Elevated IOP-induced glaucomatous damage was evaluated with TUNEL labeling of retinal cryosections from experimental 1 (n = 3) and 2 (n = 3) weeks of IOP elevation and control (n = 6) groups. TUNEL-positive cells were detected in both 1 and 2 weeks glaucomatous retinas but not in controls. The increase in the mean number of TUNEL-positive cells in the ganglion cell layer (GCL) of experimental animals from 0.5 (1 week after laser photocoagulation; 1 week vs. control, *P* = 0.035) to 3.25 (2 weeks after laser; 2 weeks vs. control, *P* = 0.036) per retinal section was indicative of progressive cell death process induced by IOP elevation (1 week vs. 2 weeks, *P* = 0.046; Fig. 1D). The percentage loss of RGCs in this experimental model was evaluated 5 weeks after IOP elevation by immunohistochemistry using antibody against Rbpms on whole mount retinas. Topographical RGC densities quantified in each of the four retinal quadrants—superior, inferior, nasal, and temporal—at 1, 2, 3, and 4 mm from the center of the optic nerve show approximately 30% of cell loss in experimental retinas (n = 8) compared to controls (n = 8): 29.4% (*P* = 0.023), 27.3% (*P* = 0.053), 26.5% (*P* = 0.061) and 30.8% (*P* = 0.022) RGC loss at 1, 2, 3 and 4 mm from the center of the optic nerve, respectively (Fig. 1E). The average RGC density in the entire glaucomatous retinas 5 weeks after IOP elevation was significantly lower than that of the contralateral controls (Fig. 1F).

**Table 1:** Average light and dark phase IOP reading before and after trabecular laser photocoagulation.

Group	Before laser				After laser					
	Light		Dark		Light			Dark		
	RE	LE	RE	LE	RE	LE	*P* value	RE	LE	*P* value
1 wk GL (n = 10)	25.9 ± 1.8	25.9 ± 2.7	36.1 ± 3.1	35.1 ± 5.2	54.6 ± 6.8	23.7 ± 2.4	< 0.001	51.4 ± 8.8	32.5 ± 4.2	< 0.001
2 wk GL (n = 10)	27.3 ± 2.3	27.5 ± 2.8	35.5 ± 2.8	36.3 ± 6.1	35.6 ± 6.1	25.9 ± 8.9	0.011	50.6 ± 6.7	30.7 ± 4.6	< 0.001

Baseline IOP readings were recorded for 1 week prior to trabecular laser photocoagulation. Significant increase in average light and dark IOP readings in the eyes at 1 week and 2 weeks after photocoagulation was noted when compared to contralateral control eyes (*P* < 0.05). All IOP readings are represented as mean ± SD. RE, right eye; LE, left eye; 1 wk GL, 1 week glaucoma; 2 wk GL, 2 weeks glaucoma.

To evaluate the extent of the DNA/RNA oxidative damage during the glaucomatous process, retinal sections from control and ocular hypertensive animals were stained with antibodies that recognize 8OHdG and 8OHG. The most prominent immunostaining was localized to cells within the GCL (Fig. 2A–C left panels). A strong reaction was also observed in photoreceptor inner segments. A relatively weak staining for 8OHdG and 8OHG compared to the GCL and photoreceptor outer segments, was present in the inner nuclear layer (INL), which contains somas of horizontal, bipolar and amacrine cells. No significant immunopositivity was observed in outer nuclear layer (ONL), which contains rod and cone photoreceptor nuclei, or in the inner plexiform layer (IPL), where dendrites of bipolar, amacrine and ganglion cells form synaptic connections. Staining in blood vessels was due to non-specific reactivity with secondary antibodies (Supplementary Fig. S1). The GCL in the mouse retina contains cell bodies of RGCs and displaced amacrine cells in an approximately 1:1 ratio (~ 45% RGCs and ~ 55% displaced amacrine cells)^27,28^. Rbpms, an established marker of RGCs^29^, was used to determine the identity of 8OHdG/8OHG-positive cells in the GCL. In both control and experimental samples, 8OHdG/8OHG staining was colocalized with Rbpms-labeled RGCs (Fig. 2A–C; some 8OHdG/8OHG/Rbpms-positive cells are pointed by yellow arrows). Very weak, if any, 8OHdG/8OHG staining was present in Rbpms-negative cells within GCL (Fig. 2B,C; white arrows). The overall pattern of 8OHdG/8OHG staining in control and glaucomatous retinas was similar, however, the intensity of immunostaining for DNA/RNA oxidative damage in RGCs was significantly increased in experimental animals. RGC immunofluorescence intensities in control and glaucomatous retinas 1 week and 2 weeks after IOP elevation (96 RGCs from each group were analyzed; n = 4 per group) were 5.56 ± 1.74, 9.24 ± 2.60 and 8.22 ± 2.69 (mean ± SD), respectively (Fig. 2D). The 1.64 fold difference between 1 week experimental and control retinas (*P* = 4.68252E−20) and 1.46 fold difference between 2 weeks experimental and control retinas (*P* = 2.50218E−12) was statistically significant. No significant difference in RGC staining intensity between 1 and 2 weeks of glaucoma was noted.

#### DNA/RNA oxidative damage is associated with mitochondria

**Figure 3:**
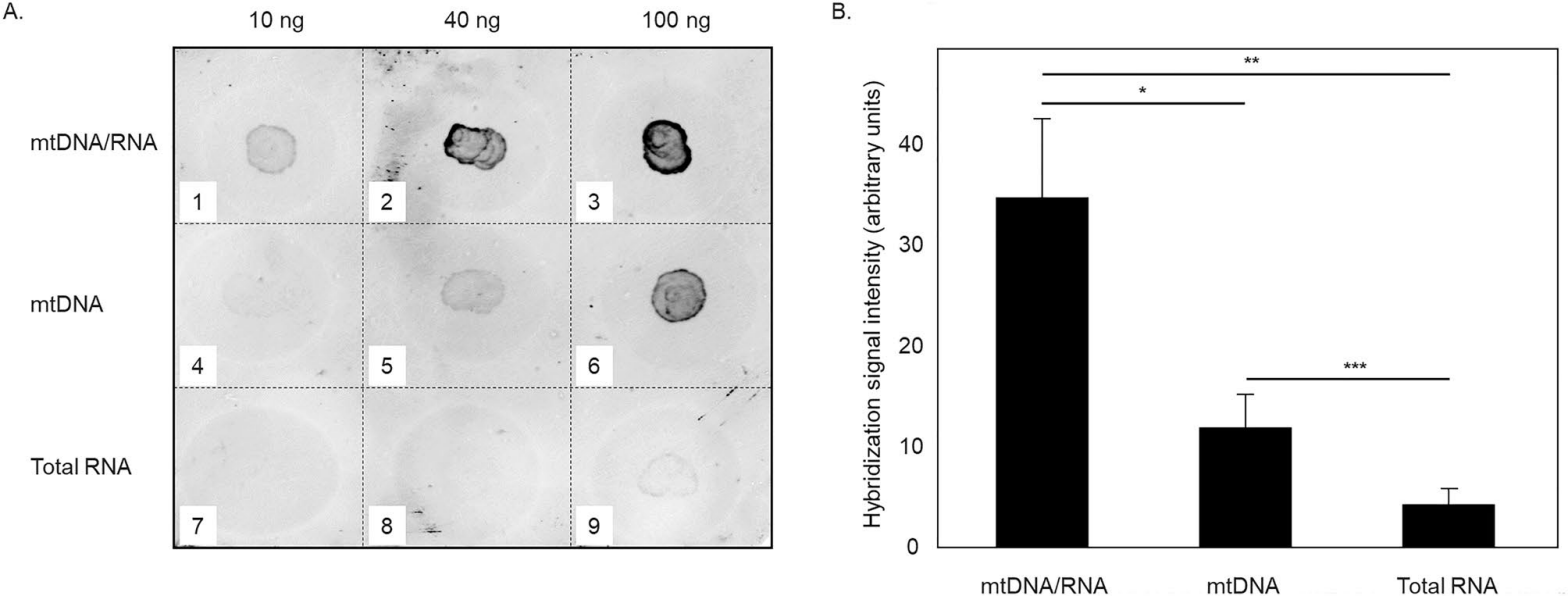
Mitochondria is the primary site of DNA/RNA oxidative damage. (**A**) Dot blot hybridization of retinal mtDNA/RNA, mtDNA (mtDNA/RNA treated with RNAse) and total RNA with antibodies against 8OHdG/8OHG. (**B**) Densitometry analysis of the dot blot hybridization indicates that the mtDNA/RNA are subjected to oxidative modification: RNA from mitochondria contributes ~ 65% to the hybridization signal and remaining 35% comes from mtDNA. mtDNA/RNA and total RNA were isolated from eight C57BL/6 control mouse retinas. Three independent hybridizations were performed. *P = 0.001, **P = 0.003 and ***P = 0.03. Data are presented as the mean ± SE.

Images presented in Fig. 2 (left panels) show that 8OHdG/8OHG staining within RGC somas is predominantly localized to the cytoplasm and to a much lesser degree to the nucleus. This suggest that the oxidatively modified DNA/RNA molecules mostly represent mitochondrial genome/RNAome or cellular RNAome. To identify which of these nucleic acids are prone to oxidative modifications, three samples, including mitochondrial DNA, mitochondrial DNA/RNA and total cellular RNA isolated from control C57BL/6 mouse retinas, were hybridized with the DNA/RNA Oxidative Damage Markers Monoclonal Antibody and the resulting hybridization signals were subjected to densitometric analysis. The strongest signal among these 3 samples was detected for mitochondrial DNA/RNA (Fig. 3A). A signal produced by this sample was clearly observed for 10 ng and its intensity increased approximately 3.5 fold for 40 ng. The signal for 100 ng was stronger than that for 40 ng. However, the increase in signal intensity was not proportional to the increase in the amount of the loaded sample, most likely due to a saturation effect. Staining for mitochondrial DNA samples (10 ng, 40 ng and 100 ng of mtDNA/RNA were treated with RNAse and spotted on the membrane) was barely detected for 10 ng, more prominent for 40 ng and much stronger for 100 ng. The signal for 100 ng of mtDNA/RNA was ~ 2.6 fold higher than that for 40 ng. With respect to total retinal RNA, no reliable hybridization signal was detected for 10 ng or 40 ng. 100 ng of loaded sample produced a faint stain with signal intensity similar to that observed for 10 ng of RNAse-treated mtDNA/RNA (Fig. 3A). Quantitative analysis of 8OHdG/8OHG in total RNA and DNA/RNA isolated from mitochondrial fraction indicates that DNA/RNA oxidative damage in the retina is taking place primarily in mitochondria (Fig. 3B). Approximately 35% of mitochondrial 8OHdG/8OHG is associated DNA and ~ 65% with the RNA.

#### Quantitative analysis of DNA/RNA oxidative damage induced by ocular hypertension

**Figure 4:**
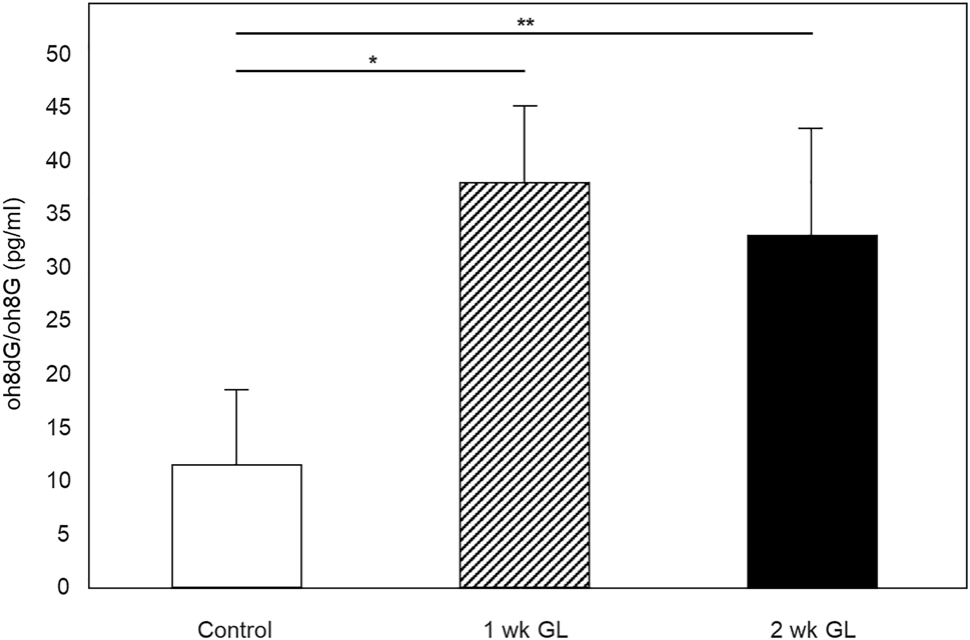
Quantitative analysis of oxidative damage to retinal mitochondrial DNA/RNA after IOP elevation. The level of 8OHdG/8OHG in mitochondrial DNA/RNA samples was measured with DNA/RNA Oxidative Damage ELISA kit. Oxidative damage to mitochondrial DNA/RNA isolated from retinas of rats with 1 and 2 weeks of elevated IOP was approximately 3.2 (n = 6, *P = 0.006) and 2.8 (n = 8, **P = 0.017) fold higher compared to controls (n = 12), respectively. Data are presented as the mean ± SD.

DNA/RNA Oxidative Damage ELISA Kit was used to measure the level of 8OHdG/8OHG in mitochondrial DNA/RNA samples isolated from control retinas and retinas of experimental animals with 1 and 2 weeks of ocular hypertension. Results of this experiment show an approximately 3.2 and 2.8 fold increase in the amount of oxidatively damaged retinal mitochondrial DNA/RNA 1 week (n = 6, *P* = 0.006) and 2 weeks (n = 8, *P* = 0.017) of IOP elevation compared to controls (n = 12), respectively (Fig. 4). ELISA data correlates with the results of immunohistochemistry that showed significantly higher staining intensity for 8OHdG/8OHG in RGCs of glaucomatous animals (both 1 and 2 weeks after IOP elevation) compared to controls.

### DNA/RNA oxidative modification in rd10 retinas with severe photoreceptor degeneration

**Figure 5:**
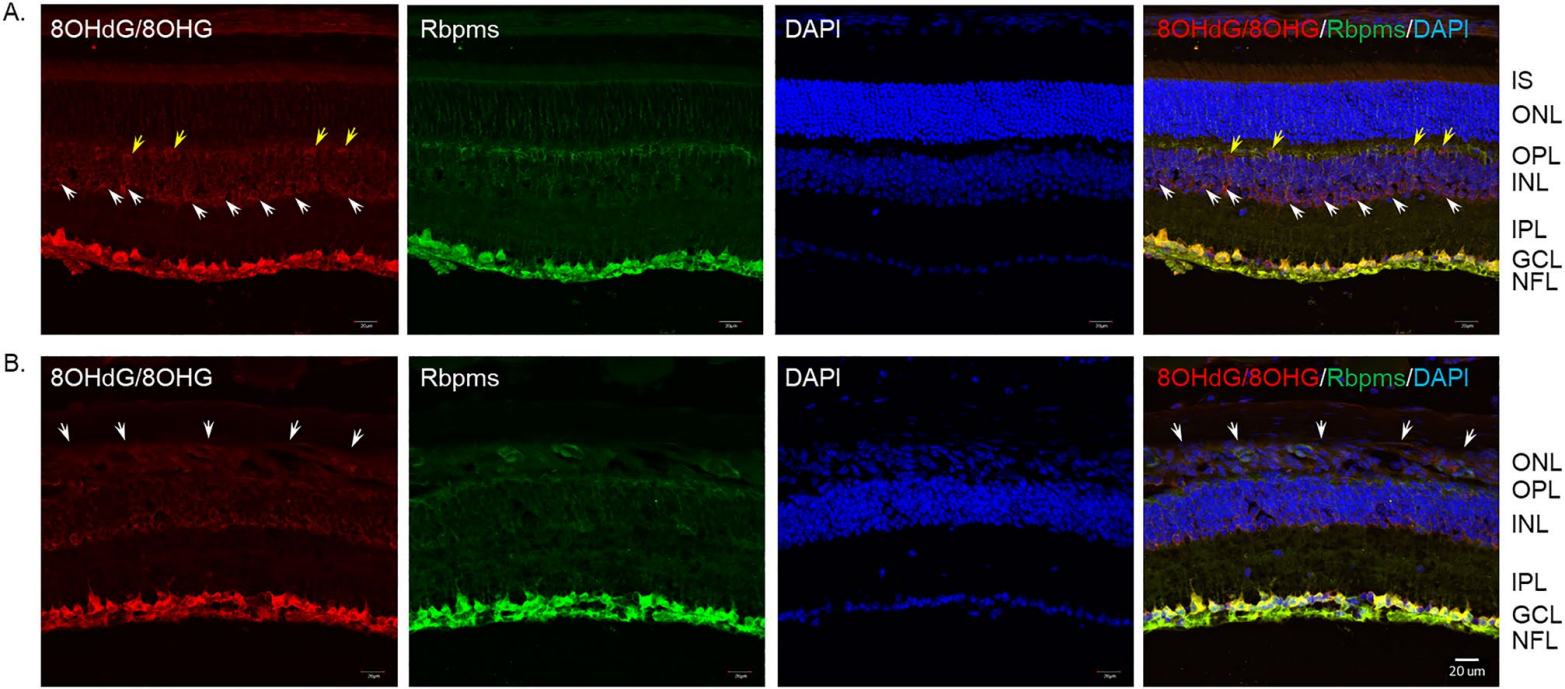
DNA/RNA oxidative damage in retinas of 2.5-month-old rd10 mouse. Representative confocal images of control (n = 3) and rd10 (n = 3) mouse retinal sections that were immunostained with antibodies for 8OHdG/8OHG. In both control and rd10 retinas, 8OHdG/8OHG staining was primarily localized to Rbpms-positive RGC somas in the GCL and RGC axons in the NFL. (**A**) In addition to RGCs, 8OHdG/8OHG relatively weak staining was present in cells within INL, particularly those adjacent to the IPL (most likely amacrine cells; white arrows) and sparsely distributed cell adjacent to OPL (most likely horizontal cells; yellow arrows). (**B**) A severe degeneration of photoreceptors and disorganization of the ONL of rd10 mouse retinas (white arrows) is present. No notable change in the pattern or intensity of the 8OHdG/8OHG staining in the GCL of rd10 mouse retinas compared to that of control was observed. DAPI was used as a nuclear counterstain. IS, photoreceptor inner segments; ONL, outer nuclear layer; OPL, outer plexiform layer; INL, inner nuclear layer; IPL, inner plexiform layer; GCL, ganglion cell layer; NFL, nerve fiber layer.

The results of retinal section staining and dot blot hybridization indicate that DNA/RNA oxidative damage observed in RGC somas occurs in mitochondria. High mitochondrial activity is necessary to support RGC functions, particularly those associated with transmission of visual information from the retina to the brain. We hypothesized that a decrease in light-evoked activity of RGCs will dampen the demand for ATP/OXPHOS, less ROS associated with this process will be produced, which in turn will lead to less DNA/RNA oxidative damage. To test this assumption, we evaluated the retinas of 2.5 month-old rd10 mice^18^. As expected, rd10 mouse retinas at this age sustained a severe degeneration of photoreceptors; a complete loss of photoreceptor IS and a dramatic thinning and disorganization of the ONL was observed (Fig. 5). 8OHdG/8OHG immunostaining in both control (Fig. 5A) and rd10 (Fig. 5B) mouse retinas was primarily localized to Rbpms-positive RGCs. However, contrary to our expectation, no significant difference was observed in the 8OHdG/8OHG staining intensity within the GCL between rd10 and control animals. A relatively weak 8OHdG/8OHG staining was observed in the INL, especially in cells adjacent to the IPL (presumably amacrine cells based on their location in the INL; Fig. 5B white arrows), as well as in sparsely distributed cells adjacent to the OPL (presumably horizontal cells based on their location and distribution in the INL; Fig. 5B yellow arrows). 8OHdG/8OHG reactivity was slightly increased in the disorganized ONL in rd10 retinas compared to that of the control ONL.

## Discussion

Oxidative stress has been commonly associated with glaucomatous damage and numerous studies, including ours, demonstrate that the alleviation of oxidative stress supports the survival of injured RGCs^16^. The current study was initiated to investigate DNA and RNA oxidative damage in retinas of animal glaucoma model. Immunohistochemical analysis of retinal sections from control animals and experimental glaucoma model 1 and 2 weeks after IOP elevation showed a strong 8OHdG/8OHG reactivity in RGCs. Since glaucomatous damage is primarily associated with the injury and subsequent degeneration of RGCs, we expected to observe an increased 8OHdG/8OHG immunoreactivity in RGCs of experimental animals. Indeed, the GCL staining intensity was higher in glaucomatous retinas 1 and 2 weeks after IOP elevation, compared to the controls. Within RGC somas, the 8OHdG/8OHG immunostaining in both control and experimental retinas was predominantly localized to the cytoplasm, suggesting that it is associated with mitochondrial (mitochondrial DNA or RNA in the mitochondria) or cellular RNA. Results of dot blot hybridization performed with 8OHdG/8OHG antibodies and total retinal RNA, retinal mtDNA/RNA or mtDNA (mtDNA/RNA was treated with RNAse to digest RNA) showed strong positive signal for the mtDNA/RNA and mtDNA, whereas the signal produced by the total retinal RNA was hardly detectable. Quantitative densitometry of these data indicate that ~ 35% of the hybridization signal for the retinal mtDNA/RNA sample is contributed by the DNA and 65% by RNA. Mitochondrial RNome is represented by the intrinsic transcriptome encoded by the mitochondrial genome, including 2 ribosomal RNAs, 22 tRNAs, 13 essential protein subunits in the OXPHOS pathway, small noncoding RNAs and the extrinsic nuclear-encoded RNA importome of some noncoding RNAs that participate in regulation of gene expression^30–32^. RNA molecules are known to be less protected against ROS under oxidative stress conditions and, therefore more susceptible to oxidation than DNA, which when damaged can be repaired by cellular DNA repair mechanisms^33,34^. The association of oxidatively modified DNA/RNA products with mitochondria explains why RGCs and photoreceptor IS are the major sites for the 8OHdG/8OHG immunoreactivity in the retina (Fig. 1). Photoreceptors and RGCs are the most metabolically active cells in the retina. In order to meet their high energy demand, these cells contain a high concentration of mitochondria^35^. More than 60% of retinal mitochondria are localized in photoreceptors, specifically in the IS of photoreceptors^36,37^. In RGCs, mitochondria are mainly distributed in the cell body^38^; however, mitochondria are also localized in dendritic synapses and branch points^39^ as well as in the axons, particularly in varicosites located in unmyelinated portions of RGC axons within the ocular globe^40^. RGCs not only have high energy demand for their normal function in the processing and transmission of visual information from the retina to the brain, but appear to be particularly susceptible to mitochondrial dysfunction. This explains a preferential loss of RGCs in the two most commonly inherited mitochondrial optic neuropathies, Leber hereditary optic neuropathy and autosomal dominant optic atrophy^41^. Furthermore, as mentioned above, mitochondrial dysfunction and the oxidative stress associated with it is currently viewed as an important factor involved in the pathophysiology of glaucoma^42^. Therefore, an increased level of 8OHdG/8OHG in retinas of an experimental glaucoma model that we observed in this study further supports the notion of oxidative stress contributing to RGC damage and degeneration.

As stated earlier, the results of immunohistochemistry show a significant increase in 8OHdG/8OHG staining intensity in RGC somas after IOP elevation compared to controls. Quantitative analysis of 8OHdG/8OHG level in mitochondrial DNA/RNA isolated from control retinas and retinas subjected to ocular hypertension with DNA/RNA Oxidative Damage ELISA indicate an approximately 3.2 and 2.8 fold higher level of oxidatively damaged mitochondrial DNA/RNA after 1 and 2 weeks of IOP elevation compared to controls, respectively. These data clearly indicate that elevation of IOP is associated with increased oxidative damage to the environment within RGC mitochondria.

Second important, in our observation from our immunohistochemistry experiment, the 8OHdG/8OHG immunoreactivity even in control animals is much stronger in RGCs than in any other cell type in the retina. The accumulation of oxidatively modified DNA/RNA molecules may result in the synthesis of defective OXPHOS constituents and, consequently lead to an increased level of ROS and decreased level of ATP production. This in turn can affect RGC normal function and lead to their damage and eventual death. Our data showing high levels of oxidatively damaged mitochondrial DNA/RNA in RGCs of control retinas suggest that oxidative stress may contribute to RGC death during normal aging, which in mice progresses at estimated rate of 2.3% per month with total loss of 41% during an 18-month lifespan and in rats at a rate of 1.5% per year with a total loss of 36% during a 24 month lifespan^43^. Loss of RGCs and their axons was also reported in humans at a rate of 0.5% per year with a total loss of 38% during a 76-year lifespan^43–45^. However, we cannot speculate about the possible involvement of oxidative damage in age related RGC loss in humans since this study did not include human tissues for analysis of oxidatively damaged RNA/DNA products.

The increase in oxidative damage to DNA/RNA in response to elevated IOP-induced damage in experimental animals was anticipated. However, it is not clear why there is a relatively high level of 8OHdG/8OHG immunoreactivity in RGCs in control retinas. Since (a) mitochondrial metabolism inevitably generates ROS and (b) 8OHdG/8OHG in control retinas is associated with mitochondrial genome/RNAome (dot blot analysis, Fig. 2), we can speculate that the observed high level of 8OHdG/8OHG immunoreactivity in RGCs in control retinas is associated with the high number of mitochondria and elevated OXPHOS activity that is necessary to support energy metabolism for the normal function of RGCs in the processing and transmission of visual information. If this assumption is correct, the level of DNA/RNA oxidative damage will be alleviated by reducing the workload of RGCs. We tested this hypothesis in rd10 mice, a model of autosomal recessive retinitis pigmentosa^18^. These mice carry a spontaneous missense mutation of the rod phosphodiesterase catalytic β subunit, which hydrolyzes cGMP in response to light. This mutation leads to rod degeneration starting around P18 followed by the death of cones. Light responses from rd10 mice can be recorded for about a month after birth. After P28, responses become sluggish and eventually undetectable^46^. Loss of photoreceptors in this model had no effect on RGC survival, dendritic architecture, anterograde axonal transport, and projections to higher visual centers in the time span from 3 to 9 months of life^47^. With respect to light-evoked RGC activity, by P60 the vast majority of RGCs did not exhibit any discrete response to the maximal intensity light flash stimuli, although a small subset of cells that remained responsive to light were noted^48^. In our experiments, we used retinas of 2.5-month old rd10 and age-matched control animals. As expected, rd10 retinas suffered a severe disorganization of the ONL associated with massive loss of photoreceptors. The INL and GCL morphology were normal. With respect to 8OHdG/8OHG immunoreactivity in RGCs, the staining intensity in rd10 and control retinas was similar. Since 8OHdG/8OHG immunoreactivity in the retina is mostly associated with oxidized RNA, one can suggest that the results observed in rd10 mouse retinas can be explained by a slow turnover of RNA molecules that were subjected to oxidation prior to severe degeneration of photoreceptors and significant loss of light-induced RGC activity. However, studies indicate that the half-life of mRNA molecules range from several minutes to several hours, with a median estimated half-life of about 7 h and that of tRNAs and rRNAs about 3 and 7 days, respectively^49–53^. This suggests that contribution of RNA that was damaged before photoreceptor degeneration to 8OHdG/8OHG immunoreactivity in RGCs of 2.5-month old rd10 mice is very unlikely. A possible explanation for the observed data is that despite our expectation, the level of mitochondrial activity in RGCs of rd10 mice even with severe degeneration of photoreceptors is similar to that of wild-type animals in order to support other cellular metabolic processes besides light-evoked activities, including spontaneous activity, which has been reported to be increased in RGCs of rd10 mice due to the loss of photoreceptors^54–56^.

In summary, the highest level of DNA/RNA oxidative damage in rodent retinas was observed in RGC. The oxidized DNA/RNA products were mostly associated with the RNA isolated from mitochondria and to a lesser degree with mitochondrial DNA. The relatively high level of 8OHdG/8OHG in RGC of wild-type animals may contribute to progressive loss of these cells during normal aging. IOP elevation in the experimental animal model causes a significant increase in the level of oxidatively damaged DNA/RNA. Oxidative damage is commonly associated with glaucoma pathogenesis; our observation provides important evidence that an elevated IOP increases the level of oxidatively damaged DNA/RNA, which may contribute to glaucomatous RGC degeneration. Finally, immunohistochemical analysis of oxidatively damaged DNA/RNA in retinas of rd10 mouse model of retinitis pigmentosa showed no significant change in the level of 8OHdG/8OHG staining in RGCs compared to the control, suggesting that the loss of light-evoked RGC activity has no significant effect on mitochondrial activity in these cells.

## Methods

### Animals and experimental glaucoma model

Adult C57BL/6 mice, rd10 mice and Brown Norway rats were used in this study. The use of animals and the procedures involving animals were approved by the Animal Research Committee of the University of California at Los Angeles and were in compliance with the National Institutes of Health Guide for the Care and Use of Animals and the ARVO Statement for the Use of Animals in Ophthalmic and Vision Research. The study was conducted in accordance with ARRIVE guidelines (https://arriveguidelines.org) for the care and use of laboratory animals. Animals were housed with standard food and water provided ad libitum in a room with the temperature set at 21 °C and illuminated with fluorescent lights (330 lx) automatically turned on at 06:00 am and off at 06:00 pm. The experimental glaucoma model was generated in the 3 month-old (250–300 g) Brown Norway rats. After 1 week of accommodation, light and dark phase IOPs were measured in awake rats with TonoLab (TonoLab; Colonial Medical Supply, Franconia, NH) twice a week. At each time point, three successful TonoLab IOP readings were recoded and averaged. Trabecular laser photocoagulation was performed as described previously^16^. Briefly, approximately 200 laser burns were delivered ab externo to the 330° trabecular meshwork at laser settings of 200 µm diameter, 100 mW power, and 50 ms durations. For IOP analysis, all IOP readings were corrected to the actual IOP and presented as mmHg. The IOP reading in the eye with experimental glaucoma was compared with the contralateral control eye of the same animal. Both, cumulative IOP elevation and the means of IOP in the dark and light phases that have been shown to be strongly correlated with RGC somal/axonal degeneration^20,57^, were analyzed in the present study. The IOP profile of every single animal using the mean of IOP at each time point was plotted. Cumulative IOP elevation was calculated by performing separate integrations of the IOP over the days of exposure for the experimental and contralateral control eye (mm Hg.days). The control eye integral value was subtracted from the experimental eye integral.

### TUNEL assay

Apoptotic cells in glaucomatous retinas were detected with the TUNEL staining. The ApopTag Red In Situ Apoptosis Detection Kit (S7165; EMD Millipore Corp., Burlington, MA) was used and the protocol provided by the manufacturer was followed. Rhodamine was used for visualization of TUNEL-positive cells and 4′,6′-diamidino-2-phenindole (DAPI) was used as nuclear stain. The quantification of TUNEL-positive cells in the GCL of each retinal section was performed in a masked fashion by a counter unaware of the experimental condition of the samples. At least 6 retinal sections from each animal were included.

### RGC quantification in retinal wholemounts

RGC quantification was performed according to the procedure described previously^29^. Briefly, deeply anesthetized animals were euthanized by inhalation of carbon dioxide followed by cervical dislocation. The eyeballs were enucleated and fixed in 4% paraformaldehyde in 0.1 M phosphate buffer for 1 h. The retinas were dissected from enucleated eyes and washed three times with PBS in triton-X (T-PBS) for 5 min. Following incubation with 10% serum for 1 h to reduce nonspecific staining, retinas were incubated with antibodies against RGC marker Rbpms^29^. Retinas were washed and then incubated with the secondary Alexa Fluor 488 goat anti-rabbit IgG antibody (1/1000) overnight at 4 °C. They were mounted flat on the glass slide with the GCL facing upward. Retinas were divided into superior, inferior, nasal, and temporal quadrants and three sampling fields (0.32 × 0.24 mm each) were imaged at 1, 2, 3, and 4 mm from the center of the optic nerve in each retinal quadrant under a fluorescence microscope (LSM410; Carl Zeiss, Oberkochen, Germany). A total of 48 images for each retina were used for the counting of Rbpms-positive cells. Cells were counted in a masked manner by a counter unaware of the experimental condition of the samples. Retinas from experimental (5 weeks after laser TM photocoagulation) and contralateral control eyes from 8 animals were used in these experiments to evaluate RGC loss induced by elevated IOP.

### Retinal sections, primary antibodies and immunohistochemistry

Eyes were enucleated, fixed with 4% paraformaldehyde and cryoprotected in 30% sucrose. 14-µm thick retinal sections were cut with cryostat. For immunohistochemistry, sections were incubated with blocking solution (20% fetal calf serum, 5% goat serum, 0.1% Triton X-100 in PBS) for 30 min and then with DNA and RNA Oxidative Damage Markers monoclonal antibodies (12,501; QED Bioscience, San Diego, CA) or with antibodies against Rbpms^29^ at 4 °C overnight. Sections were washed with 0.1% Triton X-100 in PBS and stained with donkey anti-mouse Alexa Fluor 568 (A10037; Thermo Fisher, Sherman Oaks, CA) or with donkey anti-rabbit Alexa Fluor 488 (A-21206; Thermo Fisher) at room temperature for 1 h. Sections were mounted with mounting medium containing DAPI reagent and imaged using a confocal laser scanning microscope Olympus FV3000 (Olympus, Cypress, CA). The DNA and RNA Oxidative Damage Markers monoclonal antibodies used in this study recognizes 8OHdG and 8OHG. This primary antibody has been characterized and validated earlier^58^. The expected increase in RGCs immunostaining after IOP elevation that was observed in our experiments is strong evidence for specificity of this antibody to 8OHdG/8OHG. The results of the negative control for 8OHdG/8OHG antibody are shown in Supplementary Fig. S1. Characterization and validation of the primary antibody for Rbpms was performed by our laboratory, which was the fist to identify this protein as a marker for RGCs^29^.

Quantitative analysis of RGC immunostaining for 8OHdG/8OHG in control and experimental retinas 1 and 2 weeks after IOP elevation (n = 4 per group) was performed with ImageJ software (NIH) as described previously^59–61^. Five retinal sections per retina were used to select 96 RGCs for each group of animals. The intensity of 8OHdG/8OHG-positive RGCs was estimated after background subtraction.

### Retinal mitochondria isolation, dot blot and ELISA analysis of mitochondrial DNA/RNA oxidative damage

Mitochondrial fraction was isolated from mouse or rat retinas with a Qproteome Mitochondria Isolation Kit (37,612; Qiagen, Hilden, Germany). Mitochondrial DNA/RNA from retinas was extracted using a QIAprep Spin miniprep kit (27,104; Qiagen). Mitochondria DNA isolation was confirmed by PCR with primers specific for mitochondrial genes cytochrome b (Cytb) and NADH dehydrogenase subunit 1 (ND1; Supplementary Fig. S2). To remove RNA from DNA/RNA preparation, samples were treated with RNase (11,119,915,001; Roche, Basel, Switzerland) following manufacturer’s protocol. Total retinal RNA was isolated with RNeasy Micro Kit (74,004; Qiagen). For dot blot analysis, 10 ng, 40 ng and 100 ng of mitochondrial DNA/RNA, RNase I treated mitochondrial DNA/RNA and total cellular RNA in a final volume of 10 µl were used. The samples were denatured at 95 °C for 5 min and immediately chilled on ice, loaded onto a Hybond-N nylon membrane (RPN.87N; Amersham, Amersham, UK) and covalently bonded to the membrane with UV crosslinking. The samples were incubated with DNA/RNA Oxidative Damage Markers Monoclonal Antibody (1:1000, 12,501; QED Bioscience) and then with IRDye 680RD goat-anti-mouse IgG secondary antibody (1:5000, 926-68070; LI-COR Bioscience, Lincoln, NE). The images were acquired with Odyssey CLx Imaging System (LI-COR Bioscience). Hybridization signals were quantified using the spot densitometry function of the Alpha Innotech FluorChem HD2 Gel Imaging System (Cambridge Scientific, Watertown, MA). The level of 8OHdG/8OHG in retinal mitochondrial DNA/RNA samples isolated from control and experimental animals with 1 and 2 weeks of ocular hypertension was measured with DNA/RNA Oxidative Damage ELISA Kit (589,320; Cayman Chemical, Ann Arbor, MI). The protocol provided by the manufacturer was followed.

### Statistical analysis

Data are presented as the mean ± standard deviation (SD) or standard error (SE). Student’s t-test was used to compare mean values between groups. *P* < 0.05 was considered statistically significant.

## Supplementary Information

Supplementary Information 1.

Supplementary Information 2.

## Data Availability

All data associated with this study are included in the paper.
