# Supplementary figures and images for: DNA and RNA oxidative damage in the retina is associated with ganglion cell mitochondria
Source: Sci Rep. 2022 May 24;12:8705. doi: 10.1038/s41598-022-12770-9 (PMC9130135; doi:10.1038/s41598-022-12770-9)

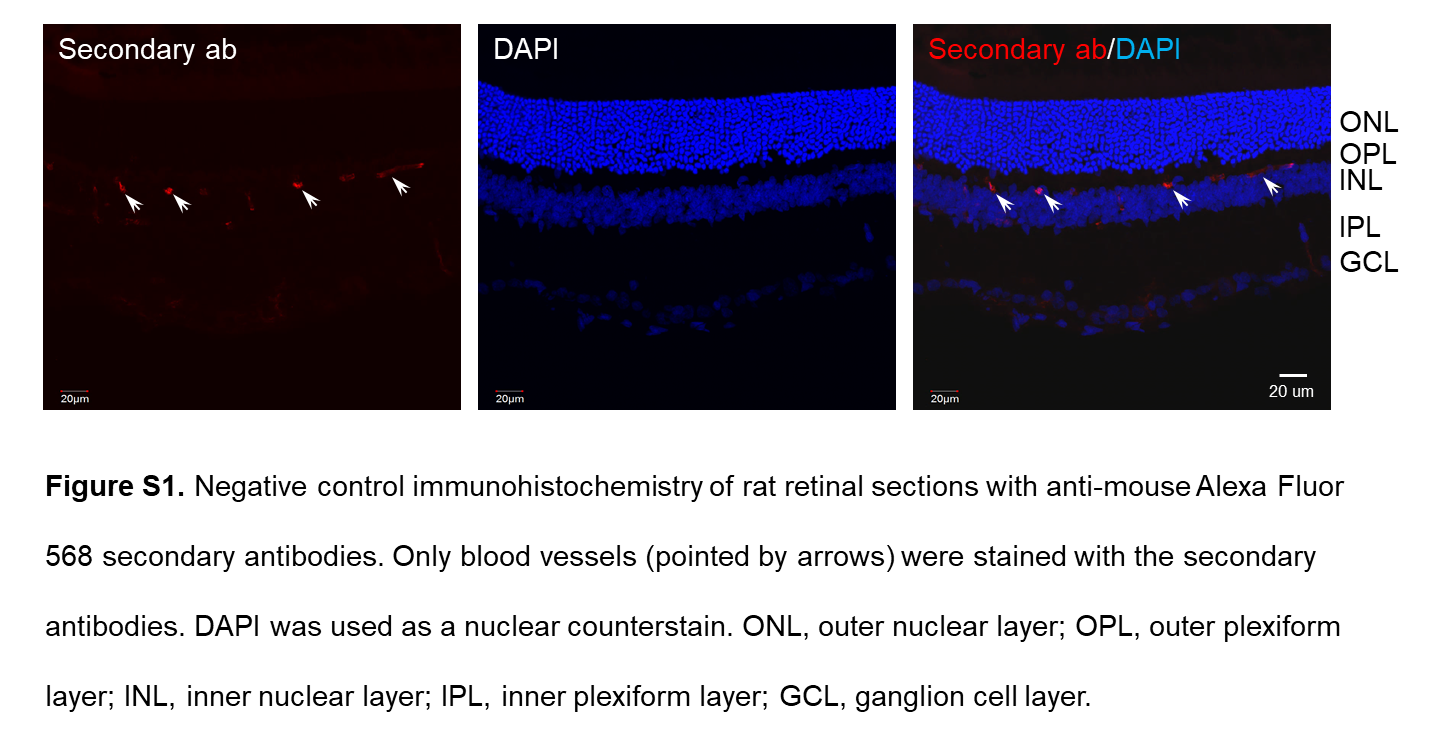

Supplement: Supplementary file 1 — Supplementary Information 1. [file 41598_2022_12770_MOESM1_ESM.tif]

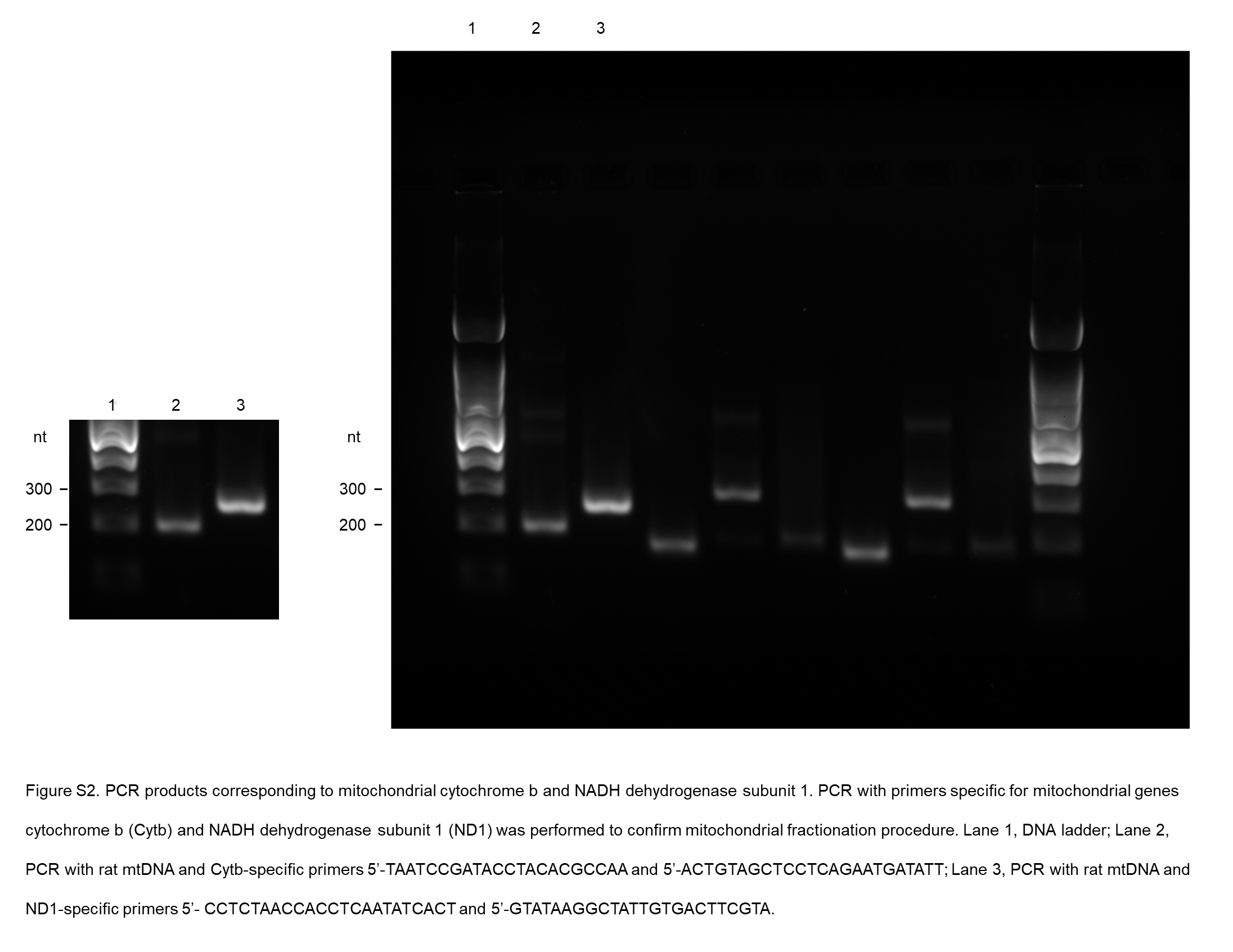

Supplement: Supplementary file 2 — Supplementary Information 2. [file 41598_2022_12770_MOESM2_ESM.tif]
